# Identifying Transcriptomic Signatures and Rules for SARS-CoV-2 Infection

**DOI:** 10.3389/fcell.2020.627302

**Published:** 2021-01-11

**Authors:** Yu-Hang Zhang, Hao Li, Tao Zeng, Lei Chen, Zhandong Li, Tao Huang, Yu-Dong Cai

**Affiliations:** ^1^School of Life Sciences, Shanghai University, Shanghai, China; ^2^Channing Division of Network Medicine, Brigham and Women’s Hospital, Harvard Medical School, Boston, MA, United States; ^3^College of Food Engineering, Jilin Engineering Normal University, Changchun, China; ^4^Bio-Med Big Data Center, CAS Key Laboratory of Computational Biology, CAS-MPG Partner Institute for Computational Biology, Shanghai Institute of Nutrition and Health, Chinese Academy of Sciences, Shanghai, China; ^5^College of Information Engineering, Shanghai Maritime University, Shanghai, China; ^6^Shanghai Institute of Nutrition and Health, Shanghai Institutes for Biological Sciences, Chinese Academy of Sciences, Shanghai, China

**Keywords:** transcriptomic, signature, classification rule, SARS-CoV-2, COVID-19

## Abstract

The world-wide Coronavirus Disease 2019 (COVID-19) pandemic was triggered by the widespread of a new strain of coronavirus named as severe acute respiratory syndrome coronavirus 2 (SARS-CoV-2). Multiple studies on the pathogenesis of SARS-CoV-2 have been conducted immediately after the spread of the disease. However, the molecular pathogenesis of the virus and related diseases has still not been fully revealed. In this study, we attempted to identify new transcriptomic signatures as candidate diagnostic models for clinical testing or as therapeutic targets for vaccine design. Using the recently reported transcriptomics data of upper airway tissue with acute respiratory illnesses, we integrated multiple machine learning methods to identify effective qualitative biomarkers and quantitative rules for the distinction of SARS-CoV-2 infection from other infectious diseases. The transcriptomics data was first analyzed by Boruta so that important features were selected, which were further evaluated by the minimum redundancy maximum relevance method. A feature list was produced. This list was fed into the incremental feature selection, incorporating some classification algorithms, to extract qualitative biomarker genes and construct quantitative rules. Also, an efficient classifier was built to identify patients infected with SARS-COV-2. The findings reported in this study may help in revealing the potential pathogenic mechanisms of COVID-19 and finding new targets for vaccine design.

## Introduction

In late 2019, the Coronavirus Disease 2019 (COVID-19) pandemic was triggered by the spread of a new strain of coronavirus named as severe acute respiratory syndrome coronavirus 2 (SARS-CoV-2). With the first confirmed case reported, the pandemic has rapidly spread all over the world, affecting 227 countries and territories. Based on the reported public health statistics from World Health Organization and Johns Hopkins University (Dong et al., 2020a), more than 32 million people were confirmed to be infected by the virus, and among them, nearly one million died. Although the outbreak of COVID-19 pandemic has been quickly and effectively controlled in several areas, the worldwide spread of COVID-19 has not been effectively controlled by all the affected countries to date. According to the summarized public health data of Sep 27, 2020, more than 9 million patients all over the world are still active (Dong et al., 2020a,b), making COVID-19 one of the most severe and long-lasting pandemics affecting human beings in the 21st century.

Given that COVID-19 triggered by SARS-CoV-2 infection is regarded as a worldwide pandemic disease, severely threatening human health, multiple studies on the pathogenesis of SARS-CoV-2 have been conducted immediately after the spread of the disease (Lv et al., 2020). For infectious diseases, two kinds of studies are conducted on different levels (Lv et al., 2020; McAloon et al., 2020): one is at the public health level, which includes the identification of pathogen, revealing the pathogen infection and transmission, and development of vaccines; the other is at the biological level, which includes revealing the biological mechanisms of pathogen infection, demonstrating the pathogenesis of infection-associated complications, and tracing the origin of the pathogen, such as in virus evolutionary studies. Although detailed biological mechanisms of SARS-CoV-2 have not been fully demonstrated, several epidemic characteristics of COVID-19 have been partially revealed, guiding the epidemic prevention of the virus at the public health level (Lv et al., 2020; Wu and McGoogan, 2020). SARS-CoV-2 spreads through two major transmission methods: direct infection *via* respiratory droplets and indirect contact *via* contaminated surfaces, especially for raw processed foods. Therefore, lockdown of epidemic areas (Inoue and Todo, 2020; Lian et al., 2020) and wearing masks (Feng et al., 2020) are necessary for the control of SARS-CoV-2 spread, which have been confirmed to be effective in China.

To date, a month after the spread of COVID-19 pandemic, the accurate detection methods of SARS-CoV-2 infection and effective infectious disease control measures, such as city lockdown and wearing masks, have slowed down the spread of the disease in certain countries and territories (Lian et al., 2020). However, the molecular pathogenies of the virus and related diseases have not been fully revealed. Given that COVID-19 is a respiratory disease, in May, a systematic transcriptomics analysis (Mick et al., 2020) about the viral pathogenic effects on the upper airway tissues attempted to reveal the biological foundations for the extremely high transmission efficacy of SARS-CoV-2 and the variable severity of clinical syndromes among infected populations. Based on such report, the suppression of innate immune responses may be one of the unique pathogenic characteristics of SARS-CoV-2 compared with other respiratory infectious diseases. Based on transcriptomics data, the authors also built effective predictive models to distinguish SARS-CoV-2 infection from other infections. Apart from this research, other studies focused on the biological and pathological effects of SARS-CoV-2, establishing an initial biological model for SARS-CoV-2 infection. Further in May, researchers from the University of Alabama at Birmingham built an interactome combining human lung-epithelial cell host interactome and SARS-CoV-2 virus interactome (Kumar et al., 2020), revealing the possible molecular mechanisms and biomarkers for COVID-19. In July, 2020, researchers from Wuhan Institute of Virology confirmed the specific role of angiotensin-converting enzyme 2 (ACE2) for SARS-CoV-2 infection and built a humanized mouse model for further studies on such virus (Jiang et al., 2020), laying a significant foundation for related studies. Similarly, researchers from Peking Union Medical College summarized the potential immune responses associated with the infection of SARS-CoV-2 and related syndromes of COVID-19 (Lin et al., 2020). Multiple studies have contributed to the revelation of the potential pathogenesis of COVID-19 and identification of new biomarkers for effective diagnosis and further vaccine development, assisting in the presentation of the deterioration of COVID-19 pandemic.

In this study, we attempted to identify new qualitative biomarkers and their quantitative rules as diagnostic models for clinical testing or therapeutic targets for vaccines design. Using the transcriptomics data of upper airway tissue from Eran’s publication (Mick et al., 2020), we integrated multiple machine learning methods to identify effective qualitative biomarkers for the distinction of SARS-CoV-2 infection from other diseases and establish quantitative rules for accurate prediction. First, two feature selection methods (Boruta (Kursa and Rudnicki, 2010) and minimum redundancy maximum relevance (mRMR) (Peng et al., 2005)) were applied on the data one by one to exclude irrelevant features and rank remaining important features in a feature list. Then, incremental feature selection (IFS) (Liu and Setiono, 1998) was applied on such list to extract biomarker genes and construct quantitative rules with the help of different classification algorithms. These identified biomarkers and rules may help in finding new targets for vaccine design and contribute to the revelation of the potential pathogenic mechanisms of COVID-19. Furthermore, an efficient classifier based on random forest (RF) (Breiman, 2001) was built, which produced the Matthew correlation coefficient (MCC) (Matthews, 1975; Gorodkin, 2004) of 0.832.

## Materials and Methods

### Gene Expression Profiles of COVID-19

We downloaded the expression profiles of 15,979 genes in 234 patients with acute respiratory illnesses (ARIs) from Gene Expression Omnibus database at^1^ (Mick et al., 2020). A total of 93 patients were infected with SARS-COV-2, 100 patients with other viruses, and 41 patients without viral infection. We aimed to identify the unique expression signature of SARS-COV-2 infection and reveal the potential pathogenic mechanisms of COVID-19.

### Boruta Feature Filtering

Boruta feature filtering (Kursa and Rudnicki, 2010; Pan et al., 2020; Yuan et al., 2020) is usually used to rapidly select all relevant features to the target labels on the basis of a random forest (RF) classifier. In brief, the calculation of Boruta includes the following steps: (1) shuffled data are created by shuffling the feature values of copies of original data; (2) RF can be trained on the original and shuffled data to measure the feature importance, and the Z score is calculated for each feature by standardizing its importance score from the RF; (3) one original feature is tagged as important when its Z score is greater than the maximum Z score of shadow features; otherwise, it is tagged as unimportant; (4) the above processes are repeated until all features are tagged as important or not.

This study adopted the program of Boruta downloaded from a public website^2^, which was implemented by python. Default parameters were used for convenience.

### Minimum Redundancy Maximum Relevance Feature Selection

Irrelevant features (genes) were excluded by Boruta method. The remaining features were further analyzed by the mRMR method (Peng et al., 2005; Wang et al., 2018; Li et al., 2019, 2020; Zhang et al., 2019; Zhang S. Q. et al., 2020; Chen et al., 2020). This method tries to find out essential features with maximum relevance to class labels and minimum redundancy to other features. The measurements to evaluate relevance and redundancy are all based on mutual information theory. The mutual information of two variables *x* and *y* can be computed by

(1) $$I\,\left(x,y\right)=\iint p\,\left(x,y\right)\,\log\frac{p\,\left(x,y\right)}{p\,\left(x\right)\,p\,\left(y\right)}\,d\,x\,d\,y,$$

where *p*(*x*) is the marginal probabilistic density of *x* and *p*(*x*,*y*) is the joint probabilistic density of *x* and *y*. Evidently, the higher the mutual information is, the stronger associations of the two variables are. The importance of a feature evaluated by mRMR is reflected by its rank in a feature list. To construct such list, mRMR method performs a loop procedure. Initially, an empty list is constructed. Features are added to such list one by one in a way that each loop determines an added feature. In each loop, for each remaining feature, calculate its relevance to class labels and mean redundancy to features already in the list. A feature with the maximum difference of relevance and mean redundancy is picked up and appended to the list. When all features are in the list, the loop stops. The obtained feature list was called the mRMR feature list in this study.

This study adopted the mRMR program downloaded from another public website^3^. Similar to the program of Boruta, default parameters were used.

### Incremental Feature Selection

By integrating a supervised classification algorithm, IFS can be used to determine the optimal number of features used to build a classifier with best performance (Liu and Setiono, 1998; Pan et al., 2020; Zhang Y.-H. et al., 2020). Based on a feature list (e.g., mRMR feature list), a series of feature subsets is produced with a step interval of one. The first feature subset consists of the top one feature in the list, the second feature subset consists of the top two features, and so on. Then, a classifier was trained on a dataset, in which samples are represented by features in each of above-constructed feature subsets. After that, the performance of each classifier was evaluated under tenfold cross-validation (Kohavi, 1995). The classifier with best performance, evaluated by MCC (Matthews, 1975; Gorodkin, 2004) in this study, can be discovered. Such classifier was called the optimum classifier. The feature subset used to construct such classifier was determined as an optimal feature subset.

### Synthetic Minority Oversampling Technique

As mentioned in Section 2.1, the used transcriptome dataset had remarkably different numbers of samples with various class labels. The largest category had 2.3 times samples as many as those in the smallest category. A classifier directly constructed on such dataset would be greatly influenced by the largest category. In view of this, the synthetic minority oversampling technique (SMOTE) approach (Chawla et al., 2002) was adopted to produce additional samples for the minor category. This approach is a type of oversampling method. For each minor category, it produces some new samples so that the minor category finally has same number of samples in the largest category. In detail, randomly select a sample in a minor category, say *x*. Compute its distance to other samples in the same category. Some samples with the smallest distances are picked up. From these samples, randomly select one sample, say *y*, and the linear combination of *x* and *y* is deemed as the new sample. Such new sample is added to the minor category. Above procedures execute several times until the predefined number of new samples have been generated. For the used dataset, each category contained 100 samples after SMOTE was applied on it. This study used the tool “SMOTE” from Weka (Frank et al., 2004; Witten and Frank, 2005), which implements the SMOTE approach.

In this study, the SMOTE approach was only used when evaluating the performance of classifiers in the IFS method. It was not used in the procedure for evaluating features.

### Classifiers

As mentioned in Section 2.4, IFS method needs a classification algorithm. This research tried four classification algorithms: (1) RF (Breiman, 2001), (2) support vector machine (SVM) (Cortes and Vapnik, 1995), (3) K-nearest neighbor (kNN) (Cover and Hart, 1967), and (4) decision tree (DT) (Safavian and Landgrebe, 1991). Their brief descriptions are as below.

#### Random Forest

Random forest is widely adopted in the investigation of biological and biomedical data (Pan et al., 2010; Zhao et al., 2018; Chen et al., 2019; Jia et al., 2020; Liang et al., 2020), and it has shown satisfactory performance in numerous studies. As a meta classifier, RF consists of multiple DTs, where each DT is learned from a bootstrap sample set with a randomly selected feature subset. For a given sample, each DT provides its prediction. RF integrates all these predictions with majority voting. In this study, we used the RF implemented in the Scikit-learn package.

#### Support Vector Machine

Support vector machine is another classic classification algorithm and also has wide applications in bioinformatics and computational biology (Chen et al., 2017; Liu et al., 2020; Zhou et al., 2020a,b). Such algorithm can deal with both linear and non-linear data. In particular, an SVM can map the nonlinear data in an original low-dimensional space to a linear data in a new high-dimensional space by a certain kernel trick. Then, the SVM attempts to detect support vectors on the margin between two classes, which consists of a hyperplane, to classify new samples. In present study, we adopted the tool “SMO” in Weka (Frank et al., 2004; Witten and Frank, 2005), which implements one type of SVM. The sequential minimal optimization algorithm (Platt, 1998) is applied to optimize the training procedures.

#### K-Nearest Neighbor

K-nearest neighbor (Cover and Hart, 1967) is a simple classification algorithm. However, in some cases, it always provides good performance. Given a test sample *s*, determination of its class consists of the following steps: (1) calculate the distances (e.g., Euclidean distance) between *s* and all samples in the training dataset; (2) find out *k* training samples with the smallest distances; (3) determine the class of *s* according to the distribution of classes of these *k* training samples, i.e., the class with the most frequency is assigned to *s*. In this study, the kNN implemented in the Scikit-learn package was adopted.

#### Decision Tree

Decision tree (Safavian and Landgrebe, 1991) attempts to supply interpretative rules in a white-box model to construct the human understanding classification or regression models. Based on a decision tree, several IF–THEN format rules can be extracted. The Scikit-learn package was applied to construct the DT classifier, depending on the CART algorithm with Gini index.

### Performance Evaluation

The MCC (Matthews, 1975; Gorodkin, 2004) was adopted to evaluate the classification performance of different classifiers. The original version was designed for binary classification problems (Matthews, 1975). As three categories were involved in this study, the multi-class version was adopted (Gorodkin, 2004), which can be calculated using the following formula:

(2) $$M\,C\,C=\frac{c\,o\,v\,\left(X,Y\right)}{\sqrt{c\,o\,v\,\left(X,X\right)\,c\,o\,v\,\left(Y,Y\right)}},$$

where matrix *X* has binary values indicating the predicted sample class, matrix *Y* also has binary values representing the true classes of all samples, and *cov*(⋅,⋅) is the covariance of two matrices. MCC has a value ranging between −1 and +1. When the classifier achieves the best performance, MCC equals +1.

Besides, the accuracy on each category and overall accuracy (ACC) were also computed, which can give full evaluation on the performance of different classifiers.

## Results

**FIGURE 1:**
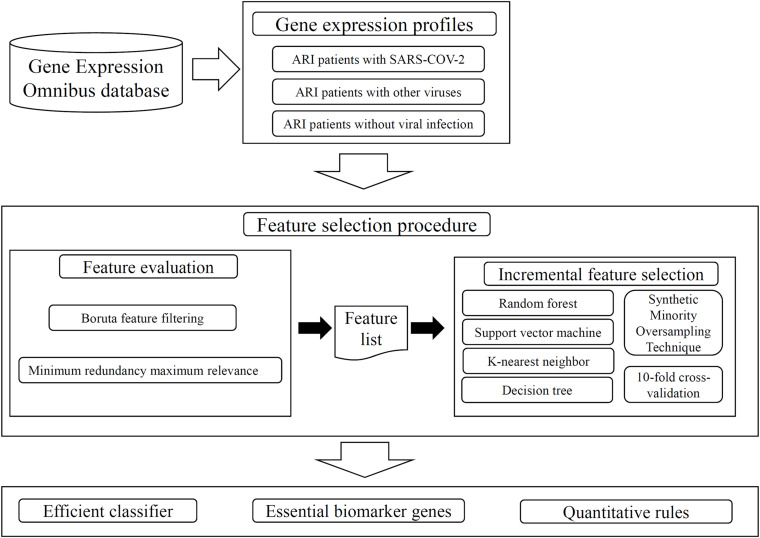
Whole procedures to analyze the gene expression profiles on ARI patients. The analyzed profiles are retrieved from Gene Expression Omnibus database. Two feature selection methods: Boruta feature filtering and Minimum redundancy maximum relevance, are applied on the profiles one by one, resulting in some important features and a feature list. The incremental feature selection method is applied on this list, which incorporates four classification algorithms, Synthetic Minority Oversampling Technique, tenfold cross-validation. An efficient classifier is constructed, essential biomarker genes and quantitative rules are extracted.

Regarding the recently reported transcriptomic data on 234 ARI patients, which included 93 patients infected with SARS-COV-2, 100 patients with other viruses, and 41 patients with no viral infection, we employed several advanced machine learning algorithms on such data. The aim was to extract essential biomarker genes and rules of SARS-COV-2. The whole procedures are illustrated in Figure 1. This section gave the detailed results.

### Results of Boruta and mRMR Methods

15,979 gene features were observed and collected in the transcriptomic data. Evidently, not all of them are related to ARI patients with SARS-COV-2 or other viruses. The Boruta approach was first applied on the transcriptomic data. 179 features were selected, which are provided in Supplementary Table 1.

179 features selected by Boruta were further analyzed by the RMR method. An mRMR feature list was obtained, which is also available in Supplementary Table 1.

### Results of IFS Method

**FIGURE 2:**
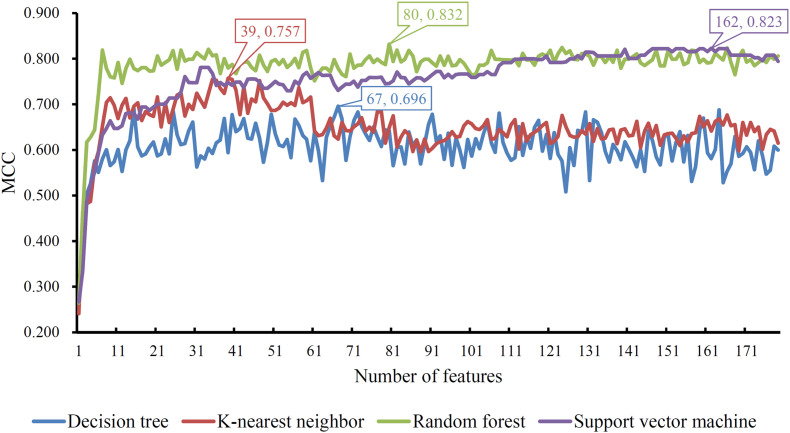
IFS curves with different classification algorithms. The random forest yields the highest MCC of 0.832 when top 80 features are used.

**FIGURE 3:**
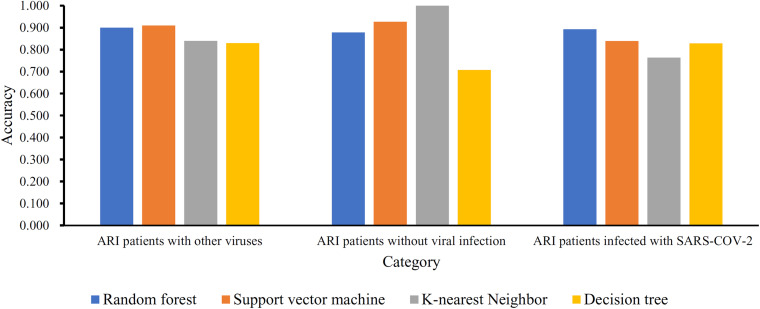
Accuracies on three categories yielded by four optimum classifiers with different classification algorithms.

Based on the mRMR feature list, IFS was carried out with the interval set to 1. 179 feature subsets were constructed. Given a feature subset and a classification algorithm, a classifier was built on samples represented by features in the subset. tenfold cross-validation was employed to evaluate the performance of each classifier. When evaluating the performance of different classifiers, SMOTE was applied to produce balance data to improve the efficiency of each classifier. The performance of different classifiers is provided in Supplementary Table 2. For an easy observation, an IFS curve was plotted with MCC as Y-axis and number of used features as X-axis, as shown in Figure 2. For RF, the highest MCC was 0.832 when top 80 features were used. Accordingly, an optimum RF classifier was built with these features. The ACC of such classifier was 0.893 (Table 1). The accuracies on three categories are shown in Figure 3, which were all close to 0.900. For other three classification algorithms, the highest MCC were 0.823 (SVM), 0.757 (kNN), and 0.696 (DT), respectively. These MCCs were obtained using top 162 (RF), 39 (kNN), and 67 (DT) features, respectively. Thus, we can build three optimum classifiers based on different classification algorithms with above-mentioned top features. The ACCs and accuracies on three categories of these classifiers are listed in Table 1 and Figure 3, respectively. Clearly, the optimum RF classifier was the best. Thus, the 80 features (genes) used to construct the optimum RF classifier were termed as optimum genes.

**TABLE 1:** Performance of the optimum classifiers with different classification algorithms.

Classification algorithm	Number of features	ACC	MCC
Random forest	80	0.893	0.832
Support vector machine	162	0.885	0.823
K-nearest neighbor	39	0.838	0.757
Decision tree	67	0.808	0.696

### Classification Rules

The optimum DT classifier used the top 67 features and yielded the MCC of 0.696. Although its performance is much lower than other three optimum classifiers, especially the optimum RF classifier, such classifier can induce several IF-THEH format rules, which can provide much biological insights to uncover the SARS-CoV-2 infection and its difference from other viral infection. Thus, we constructed a DT on all samples represented by top 67 features. Then, 24 rules were obtained, which are provided in Supplementary Table 3. Among these rules, seven rules were for the identification of ARI patients with other viral infection, eight rules were for the determination of ARI patients without viral infection and the rest nine rules were for the prediction of ARI patients with SARS-CoV-2 infection. In Section 4.2, one rule for each category would be discussed.

## Discussion

As described and summarized above, using the transcriptomics data from the upper respiratory tissues, we identified a group of potential biomarkers that can reveal the differences between SARS-CoV-2 infection and other diseases caused by viral infections, confirming the potentials of such biomarkers to contribute to the clinical diagnosis of COVID-19 and the development of new drugs/vaccines against such virus. Although current studies on COVID-19 are still limited, and many potential pathogeneses of such infectious diseases have not been revealed, all the identified biomarkers, together with related quantitative rules, are related to COVID-19-associated pathogenesis, according to recent publications.

### Qualitative Biomarkers for Distinguishing COVID-19-Infected Patients and Patients With Other Diseases

Based on our newly presented computational methods, we identified a group of significant genes that contribute to the discrimination of different infection statuses (infected by SARS-CoV-2, infected by other viruses, and disease controls). According to recent publications, some top genes participate in the discriminative biological processes of upper respiratory tissue cells under either physical or pathological conditions. Here, we analyzed the top five genes, which are listed in Table 2.

**TABLE 2:** Top five genes identified by the Boruta and mRMR method.

Rank	Ensembl ID	Gene symbol	Description
1	ENSG00000204264	PSMB8	Proteasome 20S subunit beta 8
2	ENSG00000214290	COLCA2	Colorectal cancer associated 2
3	ENSG00000147689	FAM83A	Family with sequence similarity 83 member A
4	ENSG00000108679	LGALS3BP	Galectin 3 binding protein
5	ENSG00000213928	IRF9	Interferon regulatory factor 9

The first identified biomarker was *PSMB8* (ENSG00000204264). *PSPM8* participates in the regulation of influenza virus replication in infection-associated cells such as respiratory epithelial cells (More et al., 2019); thus, this gene can distinguish cells from disease controls and from those with virus infections. Further, a research in 2020 confirmed that this gene contributes to the pharmacological regulatory effects of antimalarials against SARS-CoV-2, implying that such gene participates in the pathogenesis and related therapeutic effects of COVID-19 (Cai and Hurtado, 2020). Therefore, *PSPM8* can be regarded as a potential biomarker for distinguishing disease controls, SARS-CoV-2 infection, and other infections.

The next identified biomarker gene is *COLCA2* (ENSG00000214290), which participates in the tumorigenesis of colorectal cancer (Loo et al., 2017; Guo et al., 2018). As for the relationship between the expression of such gene and viral infection in the respiratory system, *COLCA2* has been correlated with the dysfunction of lung tissues under chronic diseases, including chronic obstructive pulmonary diseases (Verma, 2016) and chronic viral infection (Shi et al., 2019). Although many studies focused on its effective role during malignant transformation of lung cells (Peltekova et al., 2014; Noci et al., 2016; Loo et al., 2017), given the acute pathogenesis of COVID-19 (Rath et al., 2020; Rothan and Byrareddy, 2020), in upper airway tissue with chronic viral infection, and not SARS-CoV-2 infection, such gene will exhibit a unique expression level.

The next identified gene is *FAM83A* (ENSG00000147689). According to the data source (Eran’s publication) (Mick et al., 2020), this gene is a potential biomarker for identifying the affected upper airway tissues of COVID-19 patients, corresponding with our newly presented computational methods. In addition, at the biological function level, in June 2020, researchers from Turkey identified a unique expression profile of *FAM83A* in their established metabolic and protein–protein interaction networks of SARS-CoV-2-infected epithelial cells (Karakurt and Pınar, 2020), implying the specific role of *FAM83A* in distinguishing upper airway samples with SARS-CoV-2 infection from those of disease controls or with other infections.

The next genes are *LGALS3BP* (ENSG00000108679) and *IRF9* (ENSG00000213928). Both have been confirmed to be expressed in upper airway tissues (Fink et al., 2013; Clifford et al., 2016). As for their respective capacities for distinguishing upper airway samples from different subjects under various pathological conditions, *LAGLS3BP* has a specific expression level following the activation of neutrophil-mediated immune responses, which is generally observed during viral infections (Andres-Terre et al., 2015; Xu et al., 2019) including but not restricted to SARS-CoV-2 infection (Didangelos, 2020; Park et al., 2020). Therefore, *LAGLS3BP* may also help in distinguishing samples from disease controls and those from patients with viral infection but not the detailed subgrouping of infections. As for *IRF9*, according to COVID-19-related studies, similar with *LGALS3BP*, this gene is typically expressed in multiple respiratory infection diseases (Cheon et al., 2013; Wang et al., 2019). The deficiency in *IRF9* is associated with impaired control of multiple viruses (García-Morato et al., 2019), including SARS-CoV-2, building the functional relationship between *IRF9* and respiratory viral infection. Therefore, *IRF9* is a biomarker for distinguishing disease control and other virus-infected and SARS-CoV-2-infected samples.

### Quantitative Rules for Distinguishing COVID-19 Infected Patients and Patients With Other Diseases

As discussed above, we identified a group of effective biomarkers that can help in qualitatively distinguishing samples from three groups of patients. Based on recent publications, all the top features were validated to have the capacity for or participate in sample grouping at the transcriptomics level. For accurate identification of COVID-19-infected samples, we further established quantitative rules based on our newly presented computational methods, and we selected several representative rules, listed in Table 3, for each group for detailed discussion.

**TABLE 3:** Representative rules generated by DT.

Rules	Parameters	Predicted class
Rule 0	ENSG00000126709 (IFI6) ≤ 42.8197 ENSG00000100784 (RPS6KA5) > 21.5517 ENSG00000132002 (DNAJB1) ≤ 270.3345 ENSG00000111801 (BTN3A3) ≤ 36.4745 ENSG00000132600 (PRMT7) ≤ 72.2993 ENSG00000214290 (COLCA2) ≤ 57.3127 ENSG00000138755 (CXCL9) ≤ 58.9770 ENSG00000153563 (CD8A) ≤ 33.5824	ARI patients with other viral infection
Rule 1	ENSG00000126709 (IFI6) > 42.8197 ENSG00000102265 (TIMP1) ≤ 38.1346 ENSG00000133067 (LGR6) > 7.2938 ENSG00000100292 (HMOX1) ≤ 69.6592	ARI patients with SARS-CoV-2 infection
Rule 2	ENSG00000126709 (IFI6) > 42.8197 ENSG00000102265 (TIMP1) > 38.1346 ENSG00000196141 (SPATS2L) ≤ 272.6383 ENSG00000138755 (CXCL9) > 18.1848	ARI patients without viral infection

The first rule (Rule 0) involved in eight parameters, contributing to the identification of patients with virus infection but not with the SARS-CoV-2 infection. The first parameter *IFI6* (ENSG00000126709) are shown to be down-regulated in this rule, contributing to the prediction of patients with other virus infection. According to recent publications, in 2009, researchers from Duke University confirmed that *IFI6* was shown to be down-regulated during the pathogenesis of influenza and other symptomatic respiratory viral infections (Zaas et al., 2009). However, no further reports present relationships between such gene and SARS-CoV-2. *RPS6KA5* (ENSG00000100784), as the second parameter gene, has been shown to be down regulated during SARS-CoV-2 pathogenesis comparing to other virus infection. In 2017, researchers from Brazil reported that patients with Zika virus infection has specific expression level on such gene (Garcez et al., 2017), partially validating this parameter. Although no further reports confirmed the correlations between such gene and virus infection, it is still reasonable for us to regard such gene as a potential parameter for screening patients with potential viral infection. Some of other parameter genes in this quantitative rule like *DNAJB1* (ENSG00000132002), *CXCL9* (ENSG00000138755), and *CD8A* (ENSG00000153563) are well-known immune response associated proteins. As reported, *DNAJB*1 has been shown to be down-regulated during the infection of influenza A virus (Batra et al., 2016), but not SARS-CoV-2. Specifically, *CXCL9*, as a core regulator for immune responses against viral infection, has been reported to be significantly up-regulated during SARS-CoV-2 infection (Lieberman et al., 2020). Therefore, a lower expression level, which is indicated by this rule, may help us distinguish other infections from SARS-CoV-2 infection. As for *CD8A*, similar with *CXCL9*, a reversed expression level of such gene (comparing with Rule 0) during SARS-CoV-2 infection was reported (Nasab et al., 2020), helping us to build up discriminative rules for classification. Other genes like *BTN3A3* (ENSG00000111801), *PRMT7* (ENSG00000132600) and *COLCA2* (ENSG00000214290) are all proliferation associated genes, which may participate in the repair procedures after lung tissue damage caused by viral infection. The low expression level of such three genes have been reported to be associated with infection of different virus subtypes involving different tissues but not SARS-CoV-2 (Ampuero et al., 2015; Sud et al., 2018; Zhu et al., 2020), helping us to distinguish patients with/without viral infection.

As for the second rule (Rule 1) involving four parameters, such rule helps us to identify patients with SARS-CoV-2 infection. Although up to now, there are still few publications presenting the host-virus relationships specifically for SARS-CoV-2 infection, we still found strong supports for this rule. The first parameter is *IFI6* (ENSG0000 709). As we have discussed above, no direct reports confirmed that such gene has specific expression level during SARS-CoV-2 infection. However, with reverse direction of expression level, this parameter can help us to exclude patients with other common infections. As for the second and third parameter, *TIMP1* (ENSG00000102265) and *LGR6* (ENSG00000133067) has been reported to be associated with various kind of virus, including SARS-CoV-2 (Salahudeen et al., 2020; Stancioiu et al., 2020). Therefore, although they cannot help distinguishing patients with SARS-CoV-2 infection and other common virus infection, such genes can still help us to distinguish patients with SARS-CoV-2 from normal controls. For gene *HMOX1* (ENSG00000100292), researchers from University of Queensland have confirmed that the low expression level of such gene are associated with SARS-CoV-2 infection (Kumar, 2020), corresponding with this rule (Rule 1).

For the third rule (Rule 2), which help us identify patients without virus infection, four parameters were involved. The first parameter is also gene *IFI6* (ENSG00000126709). We have discussed above that it is down-regulated during other virus infection but not SARS-CoV-2 infection, which is correspondence with the expression tendency in this rule. The next gene is *TIMP1* (ENSG00000102265) which has been shown to be related to SARS-CoV-2 infection with lower expression level during pathogenesis (Salahudeen et al., 2020). In this rule for identifying patients without virus infection, such gene has been shown to be up-regulated, correspondence with previous publications. For gene *SPATS2L* (ENSG00000196141), it has been reported that such gene are up-regulated during the proliferation of B cells (Strauß et al., 2017), which is generally related to B-cell mediated humoral immunity responses. Therefore, a lower expression level of such gene as shown in this rule, may indicate no virus infection triggered abnormal inflammation. The fourth parameter gene is *CXCL9* (ENSG00000138755), which is tightly correlated with SARS-CoV-2 infection as we discussed above. The direction of such parameter is the same as its regulatory directions during SARS-CoV-2, while the absolute value of its expression level is significant lower in normal controls comparing to SARS-CoV-2 patients.

All in all, as we have discussed above, the top quantitative rules have been supported by recent publications, validating the reliability of the obtained rules.

### Comparison With the Previous Study

The COVID-19 pandemic now has turned into a world-wide pandemic. Large number of researchers from all over the world have been working on the biological and epidemic characteristic of such virus. In May, 2020, researchers from University of California, San Francisco has identified some altered gene expression patterns in the upper airway during the pathogenesis of SARS-CoV-2 (Mick et al., 2020), which can further be regarded as biomarkers for COPD at transcriptomic level. In this study, multiple biomarkers (features) were selected based on different classifier models (26 gene model, 10 gene model, and 3 gene model) using lasso method. Comparing the selected features (genes) with the optimal genes found in our study, seven genes as *TRO* (ENSG00000067445), *TIMP1* (ENSG00000102265), *IFI6* (ENSG00000126709), *LGR6* (ENSG00000133067), *WDR74* (ENSG00000133316), *IFI44L* (ENSG00000137959), and *FAM83A* (ENSG00000147689) were reported by both of two studies. As we have discussed above, genes *FAM83A*, *IFI6*, *TIMP*1, and *LGR6* have already been discussed above, confirming their significant roles for distinguishing SARS-CoV-2 and other diseases. As for *WDR74* (ENSG00000133316), *IFI44L* (ENSG00000137959), and *TRO* (ENSG00000067445), recent publications have also confirmed their correlations with SARS-CoV-2. Recently, a single-cell sequencing based analyses on the peripheral mononuclear cells identified *IFI44L* as one of the potential biomarkers to monitoring immune responses of SARS-CoV-2. As for *WDR74* and *TRO*, although no direct evidence confirmed their correlations with SARS-CoV-2, both of them have already been shown to be correlated with coronavirus infection (Mick et al., 2020; O’Brien et al., 2020), implying their potential roles during SARS-CoV-2 infection. Therefore, similar results have been identified by the previous and our studies. The shared reported genes have all been confirmed to contribute to the pathogenesis of SARS-CoV-2 and the distinction between SARS-CoV-2 infection and other virus infection involving the lung, validating the reliability of our findings.

Furthermore, this study also reported some exclusive biomarker genes compared with the previous study, such as *PSMB8*, *COLCA2*, *LGALS3BP* and *IRF9* as discussed in Section 4.1. Besides biomarker genes, our study extracted several rules, listed in Supplementary Table 3, to uncover the different expression patterns between SARS -CoV-2 infection and other viral infection or no viral infection. These rules always contain several gene parameters, which are quite complicated and can represent the patterns that the single gene cannot reflect. With different computational methods, different information about SARS-CoV-2 infection can be mined, which can all be essential parts to uncover its pathological mechanism.

## Conclusion

As discussed above, all the identified top-ranked qualitative biomarkers and quantitative rules are correlated with the identified COVID-19-associated pathogenesis and contribute to distinguishing COVID-19-infected cases from other respiratory patients with or without virus infection, validating the efficacy and accuracy of our prediction. Therefore, the application of machine learning model may efficiently assist in the identification of potential diagnostic biomarkers and candidate drug targets and help establish a standard workflow for related analyses in such field.

## Data Availability Statement

Publicly available datasets were analyzed in this study. This data can be found here: https://www.ncbi.nlm.nih.gov/geo/.

## Author Contributions

TH and Y-DC designed the study. Y-HZ, HL, and ZL performed the experiments. Y-HZ, HL, TZ, and LC analyzed the results. Y-HZ and HL wrote the manuscript. All authors contributed to the research and reviewed the manuscript.

## Conflict of Interest

The authors declare that the research was conducted in the absence of any commercial or financial relationships that could be construed as a potential conflict of interest.

## Footnotes

1 https://www.ncbi.nlm.nih.gov/geo/query/acc.cgi?acc=GSE156063

2 https://github.com/scikit-learn-contrib/boruta_py

3 http://penglab.janelia.org/proj/mRMR/

## Supplementary Material

Supplementary Table 1 List of ranked gene by mRMR.

Supplementary Table 2 Performance summary of IFS with different classifiers.

Supplementary Table 3 Rules generated by decision tree.
